# γ-Aminobutyric Acid Transporter Mutation GAT1 (S295L) Substantially Impairs Neurogenesis in Dentate Gyrus

**DOI:** 10.3390/brainsci15040393

**Published:** 2025-04-13

**Authors:** Weitong Liu, Yantian Yang, Yichen Liu, Bingyan Ni, Hua Zhuang, Kexin Chen, Jiahao Shi, Chenxin Zhu, Haoyue Wang, Jian Fei

**Affiliations:** 1 School of Life Sciences and Technology, Tongji University, Shanghai 200092, China; 1552961@tongji.edu.cn (W.L.); 2153001@tongji.edu.cn (Y.L.); 07015@tongji.edu.cn (J.S.); 2 Shanghai Engineering Research Center for Model Organisms/SMOC, Shanghai 201203, China; yantian.yang@modelorg.com (Y.Y.); bingyan.ni@modelorg.com (B.N.); hua.zhuang@modelorg.com (H.Z.); kexin.chen@modelorg.com (K.C.); guangqibingxi@outlook.com (C.Z.)

**Keywords:** GABA transporter, *Slc6a1*, neurogenesis, neurological disorders

## Abstract

**Background**: GABAergic signaling plays a crucial role in modulating neuronal proliferation, migration, and the formation of neural network connections. The termination of GABA transmission primarily occurs through the action of GABA transporter 1 (GAT1), encoded by the *SLC6A1* gene. Multiple *SLC6A1* mutations have been implicated in neurodevelopmental disorders, but their effects on the nervous system are unclear. **Methods**: We estimated the expression pattern of the GAT1 (S295L) protein using the *Slc6a1^S295L/S295L^* mouse model via RT-PCR, Western blotting, and confocal immunofluorescence. The effect of GAT1 (S295L) on hippocampal neurogenesis was investigated by neuronal marker staining (Sox2, Tbr2, NeuroD1, DCX, NeuN) and BrdU label experiments. The dendritic complexity was mapped through Sholl analysis. RNA-Seq was utilized to explore the signaling pathways and molecules associated with neurodevelopmental disorders. **Results**: We detected a remarkable decline in the quantity of type-2b intermediate progenitor cells, neuroblasts, and immature neurons in the dentate gyrus (DG) of *Slc6a1^S295L/S295L^* mice at 4 weeks. These abnormalities were exacerbated in adulthood, as evidenced by compromised dendritic length and height as well as the complexity of immature neurons. Immunofluorescence staining showed the abnormal aggregation of GAT1 (S295L) protein in neurons. RNA-seq analysis identified pathways associated with neurodevelopment, neurological disorders, protein homeostasis, and neuronutrition. The neurotrophin *Bdnf* decreased at all ages in the *Slc6a1^S295L/S295L^* mice. **Conclusions**: Our data provide new evidence that GAT1 (S295L) causes impaired neurogenesis in the DG. GAT1 mutation not only disrupts GABA homeostasis but also impairs the neurotrophic support necessary for normal hippocampal development, which may be one of the factors contributing to impaired neurogenesis.

## 1. Introduction

Neurodevelopmental disorders (NDDs) refer to neuropsychiatric conditions arising from the impaired development of the central nervous system (CNS), including attention deficit/hyperactivity disorder (ADHD), autism spectrum disorder (ASD), fragile X syndrome (FXS), Angelman syndrome, and others [1]. Patients begin to show symptoms from early childhood and may experience epilepsy and intellectual disability, which are challenging to treat. Numerous studies have indicated that a substantial proportion of genes conferring risk for NDDs are involved in encoding components of the GABAergic system [2]. GABAergic signaling is essential for regulating neuronal proliferation, guiding migration, and facilitating the establishment of neural network connections [3]. γ-Aminobutyric acid (GABA) transporter 1 (GAT1), encoded by SLC6A1, is one of such genes that have pathogenic variants. GAT1, which controls the concentration of GABA in the synaptic cleft by re-uptaking 80% of GABA into the presynaptic neurons, plays a pivotal role in fine-tuning the GABA tone in the nervous system [4].

Hundreds of the pathogenic variants of the *SLC6A1* gene have been identified [5,6,7,8,9]. Among them, GAT1 (S295L), a typical missense mutation, is associated with intellectual disability and epilepsy in patients (https://www.ncbi.nlm.nih.gov/clinvar, accessed on 28 October 2024). The mutated protein is trapped in the endoplasmic reticulum in HEK293T and iPSC cells, significantly reducing the capacity for GABA reuptake [10]. Recently, a decrease in GABA reuptake has also been demonstrated in heterozygous mouse models [11]. These lines of evidence suggest that S295L is a loss-of-function mutation of the GAT1 protein. Nevertheless, the impact of GAT1 (S295L) on the GABAergic system and the neurodevelopmental processes within the brain merit further in-depth study.

Neurogenesis is a crucial and integral process of neural development [12]. Previous studies have highlighted the role of the GABAergic system in neurogenesis within the mammalian hippocampus’s dentate gyrus (DG). The process of neurogenesis in the DG of the hippocampus exhibits numerous similarities across the broad spectrum of neural maturation, from early development to the stages of adulthood [13]. Hippocampal neurogenesis originates from neural stem cells (NSCs), transitioning through various stages, including NSC/type-1 cells, intermediate progenitor/type-2a and type-2b cells, neuroblast/type-3 cells, immature neurons, and culminating in mature granule neurons [14]. The lineage differentiation process is subject to stringent regulation and is influenced by extracellular signaling molecules. Neural stem cells express Sox2 to maintain their pluripotency and proliferative capacity [15]. Subsequently, neural stem cells differentiate into intermediate progenitor cells, marked by Tbr2 [16,17], which also express the pre-neuronal marker Ascl1 [18]. Type-2b cells initiate the expression of NeuroD1, a key transcription factor in the developmental process of the hippocampus, indicating the formation of neuroblasts. NeuroD1 is essential for the subsequent survival and maturation of neurons [19]. Type-3 cells start expressing doublecortin (DCX) and subsequently differentiate into immature neurons. These immature neurons may migrate and exit the cell cycle, ultimately maturing into NeuN-positive granule neurons. These complex neurogenic processes are regulated by various types of signals, including both the glutamatergic and GABAergic signals originating from the local neuronal networks [20]. Damage to the components of the GABA system induces alterations in neuronal plasticity, indicating that GABA plays a crucial role in brain plasticity [21]. Disruptions in the depolarizing effects of GABA impair the morphology and maturation of dendrites in the cortex [22,23] as well as decide adult quiescent neural stem cell fate [13] and the morphological development of granule neurons in the hippocampus [20,24].

In the adult hippocampus, newborn granule cells in the dentate gyrus are initially subjected to tonic activation by ambient GABA before receiving sequential innervation from GABAergic and glutamatergic synaptic inputs [20]. GABA, as the most important inhibitory neurotransmitter in the mammalian brain, initially exerts excitatory effects on newborn neurons due to the delayed expression of the potassium-chloride cotransporter KCC2 [25]. Thus, high intracellular chloride levels cause GABAergic signaling to depolarize the membrane [3,26]. The GABAergic phenotype exhibits a developmental transition from excitatory to inhibitory neurotransmission as neural stem cells (both embryonic and adult) differentiate into mature neurons, playing a crucial role in the establishment of the excitatory–inhibitory balance circuits within the nervous system.

Here, we examined the impact of GAT1 (S295L) on the development of neonatal mice and neurogenesis within the DG of the hippocampus. We observed a sharp decrease in the numbers of type-2b intermediate progenitor cells, neuroblasts, and immature neurons in homozygous mice at 4 weeks of age, indicating that the GAT1 (S295L) mutation resulted in disrupted neurogenesis within the DG. These abnormalities were exacerbated in adulthood, with DCX^+^ immature neurons exhibiting severe morphological impairments. RNA-seq analysis identified the pathways associated with neurodevelopment, neurological disorders, protein homeostasis, and protein transport. The GABA content and RNA levels of the GABA_A_ receptor were decreased at the age of 4 weeks in the hippocampus of *Slc6a1^S295L/S295L^* mice. In addition, the expression of BDNF was significantly reduced in *Slc6a1^S295L/S295L^* mice, which may be associated with the impact of neurogenesis. Our study described the neurogenesis abnormalities in the dentate gyrus caused by GAT1 dysregulation, providing new insights into the treatment of neurological disorders associated with *Slc6a1* mutations.

## 2. Materials and Methods

### 2.1. Animals

*Slc6a1^+/S295L^* mouse models were generated by Shanghai Model Organisms (Shanghai Model Organisms Center, Inc. Shanghai, China. Cat. NO. NM-KI-190014). Heterozygous, homozygous, and littermate wild-type mice were obtained by breeding heterozygous mice. All animals were kept in an SPF environment and maintained under a 12 h light/dark cycle at constant temperature (21 °C to 22 °C) and humidity (55% to 65%). Animal welfare and laboratory operations were conducted in strict compliance with the regulations on the management of experimental animals. The experimental scheme was approved by the Ethics Committee of the Shanghai Model Biology Research Center (AICUC No. 2011-0010). Body weight measurements were conducted using age-matched male and female mice to ensure consistency across groups. For immunostaining of hippocampal slices, 6–8-week-old adult male and female mice, as well as 4-week-old female mice, were used. Subsequent molecular- and protein-level analyses were performed mainly in 4-week-old female mice with a body weight range of 15–18 g at the start of the experiment. For anesthesia, mice were administered 1.25% tribromoethanol by intraperitoneal injection, followed by isoflurane inhalation anesthesia to ensure complete sedation. Cervical dislocation or other experimental procedures were performed after complete anesthesia.

### 2.2. mRNA Expression Analysis

The hippocampus or the dentate gyrus within the hippocampus was dissected in cold PBS. The total RNA of the tissues was extracted with TRIzol reagent (Tiangen, Beijing, China) according to the manufacturer’s protocol. mRNA levels were evaluated by real-time PCR using a SYBR Premix Ex Taq kit (Takara, Kusatsu, Shiga, Japan) in 384-well optical reaction plates on Applied Biosystems (ThermoFisher, Shanghai, China) following the manufacturer’s instructions. The results were normalized to *Hprt*. The relative concentration was obtained by 2^−ΔΔCT^. The primers used were as follows:
*Bdnf*rt-f:5′-TCATACTTCGGTTGCATGAAGG-3′;*Bdnf*rt-r:5′-AGACCTCTCGAACCTGCCC-3′;*Gabra1*rt-f:5′-AAAAGTCGGGGTCTCTCTGAC-3′;*Gabra1*rt-r:5′-CAGTCGGTCCAAAATTCTTGTGA-3′;*Gabra2*rt-f:5′-GGACCCAGTCAGGTTGGTG-3′;*Gabra2*rt-r:5′-TCCTGGTCTAAGCCGATTATCAT-3′;*Gabrg2*rt-f:5′-AGAAAAACCCTCTTCTTCGGATG-3′;*Gabrg2*rt-r:5′-GTGGCATTGTTCATTTGAATGGT-3′;*Hprt*rt-f:5′-CTTTGCTGACCTGCTGGATT-3′;*Hprt*rt-r:5′-TATGTCCCCCGTTGACTGAT-3′;*Slc6a1*rt-f:5′-GAAAGCTGTCTGATTCTGAGGTG-3′;*Slc6a1*rt-r:5′-AGCAAACGATGATGGAGTCCC-3′.

### 2.3. Western Blotting

The mice were sacrificed, and the brains were quickly removed on ice and stripped of the hippocampus. Proteins were extracted by RIPA lysis buffer with protease inhibitor and phosphatase inhibitor. Lysates were incubated for 30 min on ice and centrifuged at 12,000× *g* for 15 min at 4 °C. The supernatant was collected, and the protein concentration was determined using a BCA kit (Beyotime, Shanghai, China). Protein lysates were separated using 10% SDS-PAGE at 40 μg per well and transferred to a PVDF membrane (Amersham Biosciences, Piscataway, NJ, USA) after separation. The membranes were blocked with QuickBlock™ Blocking Buffer (Beyotime, Shanghai, China) for 1 h at room temperature and then incubated overnight at 4 °C with the primary antibodies against GAT1 (1:200; CST, 37342S) and GAPDH (1:5000; Beyotime, AG0122) at 4 °C overnight and incubated with HRP-conjugated goat anti-rabbit IgG (1:6000; Yamei, Wuhan, China) at room temperature for 1 h. The protein bands were analyzed with an Odyssey Infrared Imaging System (LI-COR). The optical density was quantified by ImageJ2 (2.14.0), and the relative expression of the target protein was detected with GAPDH as an internal reference.

### 2.4. Mouse Immunofluorescence

Mice were perfused with 4% paraformaldehyde (PFA) (Solarbio, Beijing, China) in PBS before dissecting the brain and post-fixed in 4% PFA at 4 °C overnight. The brain was dehydrated first with 15% sucrose and then with 30% sucrose. Using the cryostat (Leica CM1950, Wetzlar, Germany), the brains embedded in O.C.T compound (Sakura, Tokyo, Japan) were serially sectioned to a thickness of 40 μm. After washing with PBS 3 times, sections were blocked in 10% goat serum for 1 h at room temperature and incubated overnight with primary antibodies at 4 °C. The primary antibodies and dilutions used were as follows: GAT1 (1:200; rabbit-IgG; CST, Shanghai, China), Sox2 (1:400; rabbit-IgG; Beyotime, China), Tbr2 (1:2000; rabbit-IgG, Abcam, Cambridge, UK), NeuroD1 (1:200; rabbit-IgG, Invitrogen, USA), DCX (1:400; rabbit-IgG, Invitrogen, Waltham, MA, USA), and NeuN (1:200; mouse-IgG, CST, China). The secondary antibodies consisted of Alexa Fluor 488-labeled goat anti-rabbit IgG (1:1000; Beyotime, China) and Alexa Fluor Cy3-labeled Goat Anti-Mouse IgG (1:1000; Beyotime, China). Images were taken with a confocal microscope (LSM880; Zeiss, Oberkochen, Germany) fitted with individual filter sets for each channel. Image production was then performed with ImageJ2 (2.14.0).

### 2.5. Mouse Immunohistochemistry

We cut 4% paraformaldehyde (PFA)-embedded brain sections into 5 μm slices. The slides underwent deparaffinization in Roti–Histol for two 10 min intervals. Tissues fixed with PFA were processed through a graded series of ethanol solutions for dehydration, with 3 min immersions in 100%, 100%, 90%, 80%, and 70% ethanol. To demask the intracellular epitopes, the sections were subjected to fixation in citrate buffer (pH 6.0) using a microwave at 600 W for 10 min. After cooling at room temperature for 30 min, the sections were washed three times in PBS, then blocked in 3% bovine serum albumin (BSA) and 0.2% TritonX-100 in PBS for 60 min. Sections were incubated overnight with primary antibodies at 4 °C for staining with GABA. The sections were then incubated with HRP-secondary antibodies at room temperature for 1 h.

### 2.6. BrdU Labeling Experiment

For the BrdU labeling experiments, 4-week-old mice were used. Mice were given 100 mg/kg BrdU, sacrificed, and brains removed in batches after 1, 7, and 28 days.

### 2.7. Elisa

The double-antibody sandwich enzyme-linked immunosorbent assay was performed using a mouse-brain-derived neurotrophic factor (BDNF) ELISA Kit (Jianglaibio, Shanghai, China). Tissue samples, standards, biotin-labeled detection antibodies, and HRP conjugates were sequentially added to microplates pre-coated with BDNF or proBDNF capture antibodies. After the incubation and washing steps, the TMB substrate was added for color development. TMB was converted to a blue color under the catalysis of peroxidase (HRP) and then to a final yellow color under acidic conditions. The intensity of the color was positively correlated with the concentration of BDNF or proBDNF. The absorbance (OD) was measured at a wavelength of 450 nm using a microplate reader to calculate the concentration.

### 2.8. Confocal Microscopy and Image Acquisition

Confocal microscopy was performed using an inverted Zeiss laser scanning microscope (LSM880) with a 20× lens or 100× oil immersion lens. Pictures in each group were taken with the same exposure time and contrast/brightness parameters. Image production was analyzed and quantified with ImageJ2 (2.14.0). The different RGB channels were used overlapping to count the co-labeled positive cells. Mean values per sample were calculated by averaging the values of all sections of the same animal and were used to compare the cell counts of the neuronal markers of three genotypes. The experimenter was blinded to the genotypes of the samples.

### 2.9. Sholl Analysis and Three-Dimensional (3D) Reconstruction of DCX^+^ Immature Neurons

Images were obtained using a Zeiss confocal microscope (LSM880) equipped with a 100× oil immersion objective. For 3D dendritic reconstruction, confocal stack images were analyzed by the Simple neurite tracer plugin using open-access Fiji software (version 2.14.0/1.54f) [27,28]. Sholl analysis was performed using the Sholl analysis plugin (http://fiji.sc/Sholl_Analysis, accessed on 1 October 2024) based on the quantification of the number of intersections between the dendrites and the surface of the spheres in a radius increment of 5 μm. Dendrite length and height were further analyzed with Skeletonize3D (https://imagej.net/Skeletonize3D, accessed on 1 October 2024). The experimenter was blinded to the groups.

### 2.10. RNA-Seq and Data Analyses

The RNA was isolated from the hippocampus of wild-type female mice, *Slc6a1^+/S295L^* female mice, and *Slc6a1^S295L/S295L^* female mice at the age of 4 weeks, *n* = 3. Three biological replicates were taken from the experimental data. A full listing of the samples used in this analysis is provided in Supplementary Table S1. Frozen sample processing, RNA-seq, and preparatory statistical analysis were performed by Shanghai Modal Organisms (Shanghai Model Organisms Center, Inc., Cat. No. NM-KI-190014). R (4.4.0) was used to further process the data. The data were analyzed using Student’s *t*-test. Significance was defined at *p* < 0.05. Transcripts and genes were annotated using R package org.Mm.eg.db (version 3.19.1). R package tidyverse (version 2.0.0) was used to conduct statistical analysis. ClusterProfiler (version 4.12.6) was used to obtain the gene and disease ontology enrichments and pathway enrichments using the enrichGO and enrichKEGG functions with standard options. The fold enrichment score was calculated by dividing the Ratio by the BgRatio columns and taking log base 2. R package ggplot2 (version 3.5.1), pheatmap (version 1.0.12), and VennDiagram (version 1.7.3) were used to visualize the results. Results are presented as mean  ±  S.E.M. In the GO and KEGG bar plots, for each pair of comparisons, 15 representative items were manually selected from the top 60 items ranked by *p* value for visualization. In the heatmap, Minkowski distances were calculated for unsupervised clustering.

### 2.11. Statistical Analysis

Data were analyzed using GraphPad Prism v.10.0.2. Student’s *t*-test analysis was applied whenever two groups were compared. For comparisons involving more than two groups, one-way ANOVA was conducted, followed by Tukey’s multiple comparisons test as the post hoc test to determine statistical significance. For Sholl analysis, mixed-design analysis of variance (ANOVA) repeated measurements was used. Differences were considered statistically significant when *p*  <  0.05. Results are presented as mean  ±  S.E.M.

## 3. Results

### 3.1. Slc6a1^S295L/S295L^ Mice Exhibited Weight Loss and Impaired GAT1 Expression

**Figure 1:**
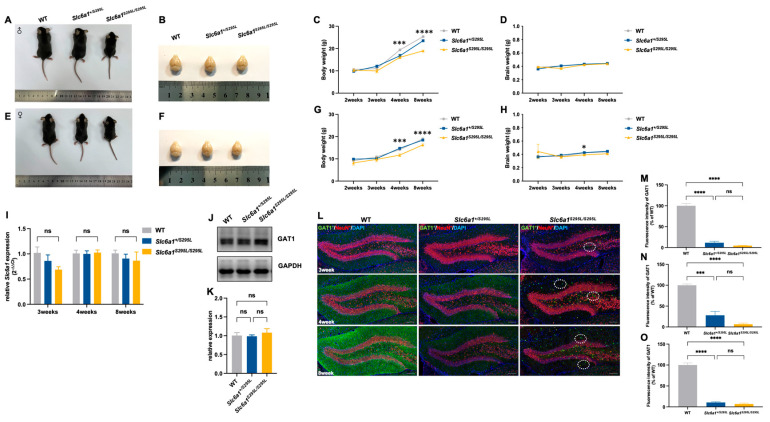
*Slc6a1^S295L/S295L^* mice exhibited weight loss and impaired GAT1 expression. (**A**,**B**) Comparison of body size and gross brain morphology among male wild-type, heterozygous, and homozygous mice. (**C**,**D**) The curves of the changes in body weight and brain weight with age of male wild-type, heterozygous, and homozygous mice (*n* ≥ 20). (**E**,**F**) Comparison of body size and gross brain morphology among female wild-type, heterozygous, and homozygous mice. (**G**,**H**) The curves of the changes in body weight and brain weight with age of female wild-type, heterozygous, and homozygous mice (*n* ≥ 20). (**I**) Expression of *Slc6a1* mRNA in the hippocampus of wild-type, heterozygous, and homozygous mice at 3 weeks, 4 weeks, and 8 weeks was detected by RT-PCR (*n* ≥ 4). (**J**) Expression of total GAT1 protein in the hippocampus of three genotypes at 4 weeks detected by Western blotting (*n* ≥ 4). (**K**) Quantitative analysis of Western blotting bands showed no significant differences in total GAT1 protein levels in the hippocampus of heterozygous and homozygous mice compared to WT (*n* ≥ 4). (**L**) Confocal images showing labeling for GAT1 (green), NeuN (red), and DAPI (blue) in the dentate gyrus of the hippocampus of three genotypes at 3 weeks, 4 weeks, and 8 weeks; 20×; scale bar, 100 μm. (**M**–**O**) Quantitative analysis of GAT1 protein fluorescence intensity in hippocampal brain slices from three genotypes of mice at the same location across different developmental stages (3 weeks, 4 weeks, and 8 weeks). * *p*  <  0.05, *** *p*  <  0.001, **** *p*  <  0.0001 vs. controls. Non-significant results were labeled as *ns* (*p* ≥ 0.05). Data represent mean  ±  S.E.M. Statistical analysis was performed using one-way ANOVA, followed by Tukey’s post hoc test.

*Slc6a1^S295L/S295L^* mice had significantly lower body weight than wild-type (WT) and heterozygous mice in the same litter from 3 weeks onward, regardless of sex (Figure 1A–H). No significant differences were observed in gross brain development or morphology among the three genotypes (Figure 1B,F). At 4 weeks, we observed a genotype- and sex-specific difference in brain weight: female *Slc6a1^S295L/S295L^* mice had significantly lower brain weight compared to WT and *Slc6a1^+/S295L^* mice (Figure 1H), whereas male mice showed no significant differences in brain weight among the three genotypes (Figure 1D). We focused our research on the female mice. We investigated the expression patterns of GAT1 (S295L) and the intracellular distribution of the protein in the hippocampus. There were no significant differences in *Slc6a1 mRNA* among the three phenotypes at different ages (Figure 1I). Western blotting analysis showed no significant differences in the total GAT1 protein levels in the hippocampus among the three genotypes (Figure 1J,K). However, we observed reduced GAT1 protein in the hippocampus of *Slc6a1^+/S295L^* mice and *Slc6a1^S295L/S295L^* mice through immunofluorescence staining (Figure 1L–O). Considering that the WB data did not indicate any differences in the total protein expression levels of the mutant GAT1, the observed reduction in the fluorescence intensity of the mutant protein detected by immunofluorescence could be attributed to alterations in protein distribution and aggregation rather than changes in overall protein abundance. By comparison, no expression of GAT1 was observed in the GAT1-knockout mice (*Slc6a1^−/−^*) (Figure S1A–C). It was noteworthy that we observed abnormal intracellular aggregation of GAT1 protein in the dentate gyrus in a small subset of three-week-old and most of the four-week-old and older *Slc6a1^S295L/S295L^* mice (Figure 1L).

### 3.2. GAT1 (S295L) Impaired Neurogenesis in the Hippocampus of Adult Slc6a1 Mutant Mice

**Figure 2:**
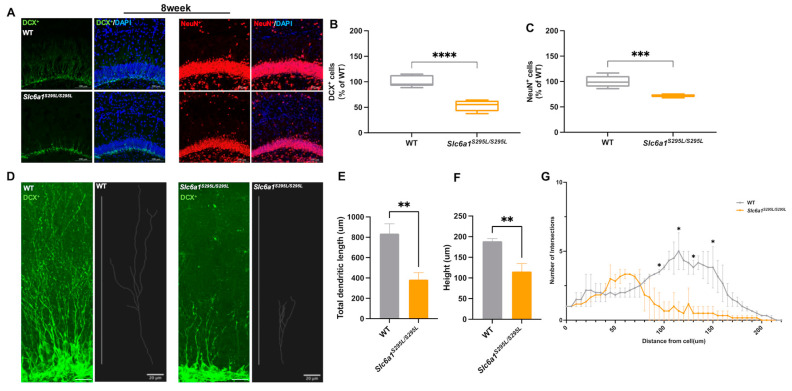
Impaired hippocampal neurons in adult Slc6a1 mutant mice. (**A**) Representative hippocampal sections of WT and *Slc6a1^S295L/S295L^* female mice at 8 weeks co-labeled with DCX and DAPI, NeuN and DAPI: 20×; scale bar, 20 μm. (**B**) DCX-positive cells in the sum of the DG compared to the female WT group (100% grey bars) (*n* ≥ 4). (**C**) NeuN-positive cells in the sum of the DG in comparison to the female WT group (100% grey bars) (*n* ≥ 4). (**D**) Representative confocal images and 3D reconstruction of DCX staining in the DG of 8-week-old WT and *Slc6a1^S295L/S295L^* mice: 100× oil; scale bar, 20 μm. (**E**,**F**) DCX^+^ immature neurons exhibited significantly lower total dendritic length and height in *Slc6a1^S295L/S295L^* mice than the WT (*n* ≥ 3). (**G**) Sholl analysis of DCX^+^ immature neurons between WT and *Slc6a1^S295L/S295L^* mice at 8 weeks (*n* ≥ 3). * *p*  <  0.05, ** *p*  <  0.01, *** *p*  <  0.001, **** *p*  <  0.0001 vs. controls. Data are presented as mean  ±  S.E.M. Statistical analysis was performed using Student’s *t*-test.

The abnormal expression patterns of GAT1 (S295L) prompted us to investigate the impact of GAT1 dysregulation on the hippocampal neurons of adult mice. To achieve this, we simultaneously assessed the number of DG neurons in the adult male and female WT and *Slc6a1^S295L/S295L^* mice. Strikingly, we observed a significant depletion of DCX (doublecortin)-positive immature neurons and a considerable decrease in NeuN-positive post-mitotic mature neurons in the adult female *Slc6a1^S295L/S295L^* mice compared to the WT controls (Figure 2A–C). In contrast, no significant differences were detected in the male mice (Figure S1D,E). Given that the female *Slc6a1^S295L/S295L^* mice exhibited abnormal brain weight loss, we focused on the development of the hippocampal neurons in the female mice. Furthermore, we conducted a 3D reconstruction analysis of the DCX-positive cells in 8-week-old female WT and *Slc6a1^S295L/S295L^* mice (Figure 2D). A pronounced reduction in the total dendritic length and height of the DCX-positive neurons was noted in the *Slc6a1^S295L/S295L^* mice (Figure 2E,F), indicating neurogenesis impairment of the dentate gyrus neurons in the *Slc6a1^S295L/S295L^* mice. Sholl analysis demonstrated diminished overall dendritic complexity, reflected by a decrease in the number of dendritic intersections of the DCX-positive cells within the DG of the *Slc6a1^S295L/S295L^* mice (Figure 2G). Notably, a significant reduction in dendritic crossings at 100–150 μm from the soma was observed for the DCX^+^ cells in the *Slc6a1^S295L/S295L^* mice (Figure 2G). The observed decrease in dendritic intersections suggested a disruption of the structural maturation of the DCX-positive immature neurons in the DG. Since dendritic arborization is critical for the integration of synaptic inputs and the establishment of functional neural circuits, these findings implied that the GAT1 (S295L) mutation compromised not only the structural intricacy but also the functional capacity of immature neurons. Such deficits in dendritic architecture may underlie the impaired neurogenesis and the associated functional consequences in Slc6a1^S295L/S295L^ mice, further supporting the role of Slc6a1 in maintaining neuronal development and connectivity. To rule out whether the GAT1 protein aggregation observed in *Slc6a1^S295L/S295L^* mice (Figure 1L) was associated with the neuronal damage, we collected 6-week-old adult *Slc6a1^−/−^* mice and compared the developmental status of the DCX-positive neurons in the hippocampus with that of age-matched *Slc6a1^S295L/S295L^* mice (Figure S2A). In the DG of *Slc6a1^−/−^* mice, the number of DCX^+^ neurons was significantly reduced compared to the wild-type mice (Figure S2B). Additionally, the dendritic length and height were markedly decreased (Figure S2C,D), and Sholl analysis revealed a significant reduction in dendritic branching complexity (Figure S2E). However, no significant differences were observed between the *Slc6a1^S295L/S295L^* mice and *Slc6a1^−/−^* mice. The findings implied that the impacts of Slc6a1 knockout and S295L mutation on neurogenesis and dendritic morphology were likely comparable. It appeared that the impairments in neuronal development mainly stemmed from the loss of GAT1 function rather than the aggregation of mutant proteins induced by the S295L mutation.

### 3.3. GAT1 (S295L) Impaired the Neuronal Fate Choices in Neurogenesis in the DG of Mutant Mice

**Figure 3:**
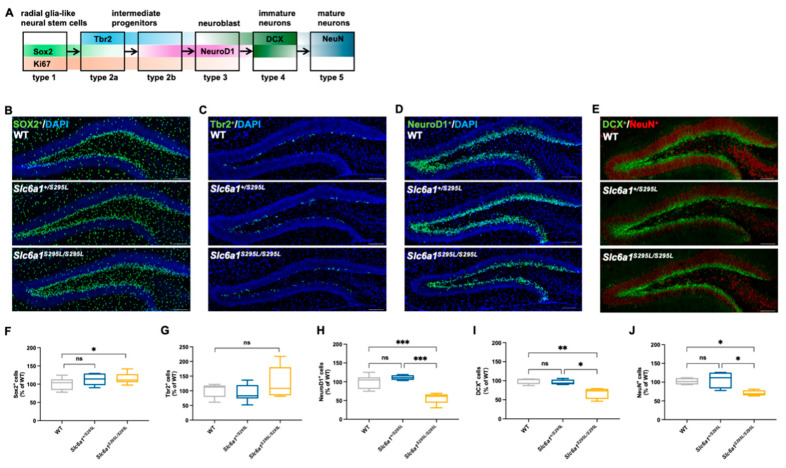
GAT1 (S295L) impaired neurogenesis in the DG. (**A**) Schematic diagram of neurogenesis in the dentate gyrus. (**B**) Representative hippocampal sections of WT, *Slc6a1^+/S295L^* mice, and *Slc6a1^S295L/S295L^* mice at 4 weeks co-labeled with Sox2 and DAPI: 20×; scale bar, 100 μm. (**C**) Representative hippocampal sections of the three genotypes at 4 weeks co-labeled with Tbr2 and DAPI. 20×, scale bar, 100 μm. (**D**) Representative hippocampal sections of the three genotypes at 4 weeks co-labeled with NeuroD1 and DAPI: 20×; scale bar, 100 μm. (**E**) Representative hippocampal sections of the three genotypes at 4 weeks co-labeled with DCX and NeuN: 20×; scale bar, 100 μm. (**F**) Sox2-positive cells in the sum of the DG in comparison to the WT group (100% grey bars) (*n* ≥ 3). (**G**) Tbr2-positive cells in the sum of the DG in comparison to the WT group (100% grey bars) (*n* ≥ 3). (**H**) NeuroD1-positive cells in the sum of the DG in comparison to the WT group (100% grey bars) (*n* ≥ 3). (**I**) DCX-positive cells in the sum of the DG in comparison to the WT group (100% grey bars) (*n* ≥ 3). (**J**) NeuN-positive cells in the sum of the DG in comparison to the WT group (100% grey bars) (*n* ≥ 3). * *p*  <  0.05, ** *p*  <  0.01, *** *p*  <  0.001 vs. controls. Non-significant results were labeled as *ns* (*p* ≥ 0.05). Data are presented as mean  ±  S.E.M. Statistical analysis was performed using one-way ANOVA, followed by Tukey’s post hoc test.

**Figure 4:**
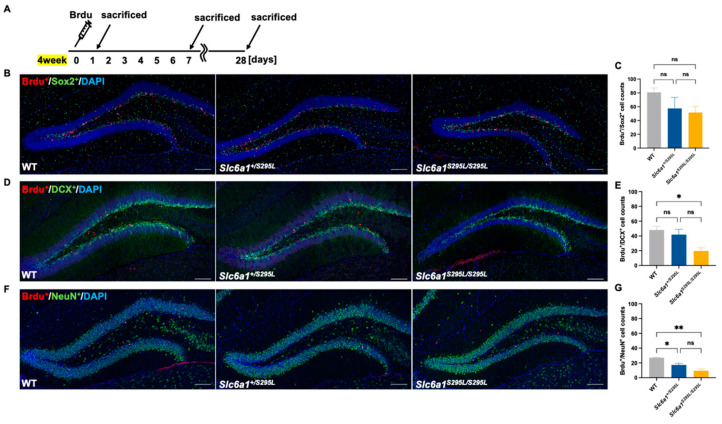
Neurogenesis in the DG was affected by GAT1 (S295L). (**A**) Experimental paradigm assessing the proliferation of neurons at the age of 4 weeks. (**B**) Representative hippocampal sections of WT, *Slc6a1^+/S295L^* mice, and *Slc6a1^S295L/S295L^* mice co-labeled with BrdU and Sox2 (day 1): 20×; scale bar, 100 μm. (**C**) Co-labeling with BrdU and Sox2 in the neuron counts of the DG among the three genotypes (*n* ≥ 3). (**D**) Representative hippocampal sections of WT, *Slc6a1^+/S295L^* mice, and *Slc6a1^S295L/S295L^* mice co-labeled with BrdU and DCX (day 7): 20×; scale bar, 100 μm. (**E**) Co-labeling with BrdU and DCX in the neuron counts of the DG among the three genotypes (*n* ≥ 3). (**F**) Representative hippocampal sections of WT, *Slc6a1^+/S295L^* mice, and *Slc6a1^S295L/S295L^* mice co-labeled with NeuN and BrdU (day 28). (**G**) Co-labeling with BrdU and NeuN in the neuron counts of the DG among the three genotypes (*n* ≥ 3). * *p*  <  0.05, ** *p*  <  0.01, vs. controls. Non-significant results were labeled as *ns* (*p* ≥ 0.05). Data are presented as mean  ±  S.E.M. Statistical analysis was performed using one-way ANOVA, followed by Tukey’s post hoc test.

Considering that the adult *Slc6a1^S295L/S295L^* mice demonstrated a tendency towards impaired DCX^+^ neurons, we focused on the influence of GAT1 (S295L) on the neurogenesis in the dentate gyrus (Figure 3A). To explore the effect of GABAergic signaling on early progenitor cells at the onset of neuronal cell lineage development, we carried out Sox2 (a marker expressed by NSCs) staining of brain sections obtained from the WT, *Slc6a1^+/S295L^*, and *Slc6a1^S295L/S295L^* mice at 4 weeks. GAT1 (S295L) did not significantly impact the number of Sox2-stained cells (Figure 3B,F). Notably, there was a significant increase in Sox2^+^ cells in the *Slc6a1^S295L/S295L^* mice at 4 weeks (Figure 3F). Moreover, no significant alteration was detected in the quantity of the Tbr2-stained type-2 cells in these mice (Figure 3C,G). These findings suggested that *Slc6a1^+/S295L^* and *Slc6a1^S295L/S295L^* mice still maintained a relatively normal early progenitor cell pool, indicating GAT1 (S295L) did not impair the proliferation of early progenitor cells in the DG. Intermediate progenitor cells make the neuronal fate choices that give rise to neuroD1-positive type-2b cells and neuroblast-like type-3 cells [29]. In the DG of the *Slc6a1^S295L/S295L^* mice, the number of NeuroD1-positive cells was significantly decreased (Figure 3D,H), indicating that GAT1 dysregulation began to exert an influence at the progenitor cell stage in the dentate gyrus. Consistent with the aforementioned results, in 4-week-old *Slc6a1^S295L/S295L^* mice, we also observed a marked decrease in the number of DCX-labeled immature neurons and NeuN-labeled mature neurons (Figure 3E,I,J); this indicated that GAT1 (S295L) impaired the development of the neuroblasts and the maturation of the neurons. To further determine whether the GABAergic inputs could block the cell-cycle progression in the DG, we subsequently labeled dividing cells with a single injection of BrdU (Figure 4A). The quantification of BrdU^+^/Sox2^+^ co-labeled type-1 cells revealed no statistically significant differences among the three genotypes (Figure 4B,C), suggesting that the proliferation of the neural stem cells was not directly affected by the GAT1 (S295L) mutation. However, we observed a significant reduction in the number of immature neurons co-labeled with BrdU and DCX, as well as mature neurons co-labeled with BrdU and NeuN, in *Slc6a1^S295L/S295L^* mice at both 7 days and 28 days post-injection of BrdU at the age of 4 weeks (Figure 4D–G). These findings indicated that the GAT1 (S295L) mutation disrupted the subsequent stages of adult neurogenesis, particularly the transition from type-2a progenitor cells to type-2b intermediate progenitors. This disruption likely reflected impaired maturation and differentiation processes, which ultimately affected the generation of both immature and mature neurons in the adult brain.

### 3.4. Transcriptome Analysis Provided Further Evidence of Role of Slc6a1 (S295L) Mutation in Neurodevelopmental Disorders

**Figure 5:**
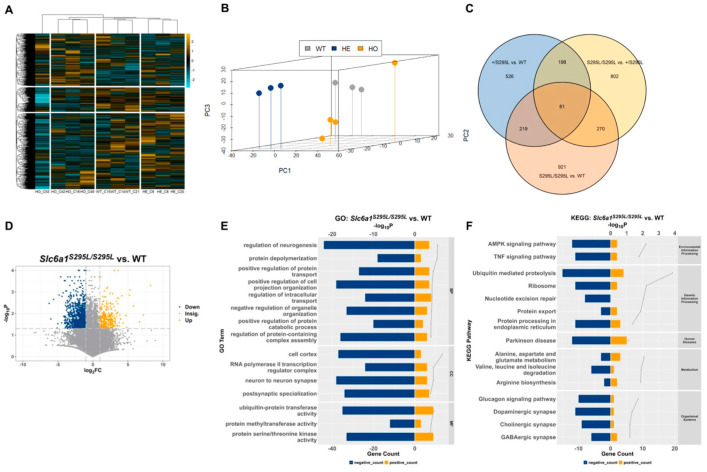
Analysis of differentially expressed genes (DEGs) in transcriptomic profiles of the hippocampus of WT and *Slc6a1* mutant mice at 4 weeks. (**A**,**B**) Heat map and clustering of Z-score-normalized principal component analysis (PCA) of gene expression data. (**C**) Venn diagram showing DEG overlaps. (**D**) Volcano plots used for DEG analysis in *Slc6a1^S295L/S295L^* vs. WT (|log_2_(*FoldChange*)| ≥ 1). (**E**) Representative results of GO enrichment analysis. (**F**) Representative results of KEGG enrichment analysis. Downregulated gene counts were marked as “negative” after the enrichment analysis. *p* < 0.05, statistical analysis was performed using Student’s *t*-test.

To gain insights into the transcriptional changes associated with *Slc6a1* mutation phenotypes, we performed RNA-seq of the hippocampus from the three female genotypes at 4 weeks. The quality control based on the unsupervised clustering and principle component analysis (PCA) of the biological repeats indicated that the transcriptome data were reliable (Figure 5A,B). RNA-seq data analysis identified 409 significantly upregulated genes and 415 significantly downregulated genes in *Slc6a1^S295L/S295L^* vs. WT (*p* < 0.05, |log_2_(*FoldChange*)| ≥ 1), (Figure 5C,D). Gene Ontology (GO) functional analysis of these DEGs showed enrichment in functions related to cell migration, neurodevelopment, synapse structure, calcium-related cell functions, protein processing, degradation, and transportation (Figure 5E, Supplementary Table S1). Corroborating the GO analysis results, the KEGG pathways enriched in the DEGs included pathways related to neurodevelopment, neurological disorders, protein homeostasis, protein transportation, important signaling pathways, etc. (Figure 5F, Supplementary Table S1). These results were consistent with our observations and provided further evidence supporting the hypothesis that the damaged GAT1 transportation and subcellular localization were the main factors influencing the GABA reuptake disorders.

Notably, we observed a significant downregulation of neurotrophin expression in the RNA-seq results. Brain-derived neurotrophic factor (BDNF), known for its role in modulating structural plasticity and dendritic spine maturation [30], was found to have significantly decreased mRNA levels in the DG of 2-week-, 3-week-, 4-week-, and 8-week-old *Slc6a1^S295L/S295L^* mice (Figure S3A), aligning with the observed deficits in the development of the neurons. The content of BDNF was significantly decreased in both the heterozygous and homozygous mice at 4 weeks as detected by ELISA (Figure S3B), while there was no difference in the levels of proBDNF, which is the precursor of BDNF, observed in the mutant mice (Figure S3C). In addition, we observed a downregulation of GABA_A_ receptor expression in the RNA-seq results. We hypothesized that disruptions in GABA signaling played a crucial role in the abnormal neurogenesis of the dentate gyrus. To investigate the relationship between the *Slc6a1* mutation and GABAergic signaling, we compared the differences in the expression of the components of the GABA_A_ receptors in the DG of wild-type mice, *Slc6a1^+/S295L^* mice, and *Slc6a1^S295L/S295L^* mice at 4 weeks. Consistent with the transcriptomic findings, we found a significant downregulation of multiple GABA_A_ receptor subunits, including *Gabra1*, *Gabra2*, and *Gabrg2* at the mRNA level at 4 weeks (Figure S3D–F). In addition, we examined the protein expression changes of some GABA_A_ receptor subunits by WB and found that the GABRA2 protein was significantly downregulated in the hippocampus of the *Slc6a1^S295L/S295L^* mice, while GABRA5 and GABRG2 did not change significantly (Figure S3G–I). Overall, we propose that the inability of GAT1 to effectively manage the excess GABA in the synaptic cleft leads to an adaptive reduction in the transcriptional levels of multiple GABA_A_ receptor subunits.

## 4. Discussion

In the present study, we investigated the relationship between the S295L mutation of the GAT1 protein and its impact on adult neurogenesis. Our results showed that *Slc6a1^S295L/S295L^* mice lost weight since the age of 4 weeks. In addition, *Slc6a1^S295L/S295L^* mice exhibited severe impairment of neurogenesis within the DG of the hippocampus. Our results showed a significant decline in the type-2b intermediate progenitor cells, neuroblasts, and immature neurons in the homozygous mice, accompanied by a decrease in newly mature neurons. This finding underscored that the GAT1 (S295L) mutation disrupted the neurogenesis in the hippocampal dentate gyrus. The severity of these phenotypes became markedly more pronounced by the eighth week, characterized by diminished dendritic length and height as well as complexity in immature neurons. Our study identified that GAT1 dysregulation induced dentate gyrus dysplasia, providing a new perspective for the treatment of GAT1-mutation-related neurological disorders.

Previous studies have demonstrated that mutations in the *Slc6a1* gene are strongly associated with epilepsy and cognitive impairments. *Slc6a1^+/S295L^* mice exhibited spike-wave discharges (SWDs) and motor arrest, which were consistent with the absence seizures observed in humans [31]. Furthermore, homozygous *Slc6a1^S295L/S295L^* mice showed severe behavioral and EEG abnormalities, including heightened epileptic activity and deficits in motor coordination and cognitive function [32]. These findings underscore the critical role of *Slc6a1* in maintaining normal neuronal function. Our observed developmental defects in the hippocampal neurons aligned with these behavioral manifestations in mice, providing further evidence of the impairment of neurodevelopment by abnormalities in *Slc6a1*.

The precursor stem cells in the dentate gyrus of the hippocampus maintained normal quantity and status (Figure 3B,C,F,G). However, there was a sharp decrease in the number of type-2b and type-3 cells (Figure 3D,I) in the mutant mice, suggesting that the GAT1 mutation impacted the differentiation process from type-2 to type-3 cells. Previous studies have shown that GABAergic inputs depolarized type-2 cells, which initiated an increase in Ca^2+^ and the expression of NeuroD [24]. Consistent with this study, we observed a downregulation of a series of calmodulin-related genes in our RNA-seq analysis, along with a significant downregulation of the RNA levels of GABA_A_ receptor subunits. We suspected that the loss of function of the GAT1 (S295L) protein led to a temporary increase in the GABA concentration in the synaptic cleft. The excess GABA caused the adaptive downregulation of the GABA_A_ receptors, ultimately resulting in reduced GABA synthesis and transport. Another question is whether the reduction in type-3 cells is due to an upstream differentiation blockade or whether neuroblasts undergo premature apoptosis. Additionally, RNA-seq analysis revealed an upregulation of apoptosis-related genes in the DG of the *Slc6a1^S295L/S295L^* mice. The isolation of specific neurons to verify the process of differentiation is necessary in the future.

Our findings revealed a significant impairment in neurogenesis due to *Slc6a1* mutation, accompanied by a notable decrease in the levels of brain-derived neurotrophic factor (BDNF). BDNF is a key player in promoting neuronal survival, growth, and synaptic plasticity [33]. The decrease in BDNF expression in the *Slc6a1^S295L/S295L^* mice further underscored the importance of GABA signaling in modulating neurotrophic support. It has been established that GABAergic synaptic activity in the developing hippocampus induces BDNF secretion and promotes the maturation of perisomatic GABAergic synapses [34]. As BDNF secretion might also promote the maturation of nearby synapses, there may be bidirectional crosstalk with abnormal GABA signaling and reduced BDNF. The impaired GABA signaling resulting from the *Slc6a1* mutation may lead to insufficient BDNF release, thereby compromising the neurogenic process and contributing to deficits in hippocampal neurogenesis. Moreover, the interaction between GABA and BDNF may create a feedback loop, where reduced BDNF levels further exacerbate GABAergic dysfunction, leading to a vicious cycle that impairs neurogenesis.

Considering the potential protein aggregation detected in the brain slices obtained from the homozygous *Slc6a1^S295L/S295L^* mice (Figure 1L), we suspected that the GAT1 (S295L) protein exerted a certain level of proteotoxicity. The comparison between the *Slc6a1^−/−^* mice and *Slc6a1^S295L/S295L^* mice provides compelling evidence that the loss of GAT1 function is a critical factor contributing to neurodevelopmental impairments (Figure S2A–E). However, whether the possible aggregation of GAT1 (S295L) contributes to additional cellular damage beyond the loss of GAT1 function is uncertain. Specifically, the S295L mutation likely induces abnormal protein aggregation, which could lead to cellular stress responses such as endoplasmic reticulum (ER) stress or impaired autophagy. These additional cellular stressors may exacerbate neurodevelopmental damage. It has been well documented that protein aggregation is associated with neurodegenerative diseases, including Alzheimer’s and Parkinson’s diseases, and contributes to cellular toxicity. The RNA-seq analysis indicated that protein homeostasis was disrupted in the *Slc6a1^S295L/S295L^* mice. Whether the disease phenotypes caused by GAT1 (S295L) are entirely due to the loss of protein function is worthy of further in-depth investigation. In addition, GAT1 expression was significantly diminished with abnormal aggregation in the PV-positive neurons of the homozygous mice (Figure 1L and Figure S4). We suspected that the aberrant GAT1 expression in the PV-positive neurons elicited abnormal GABAergic signaling, which could impair the differentiation of type-2 to type-3 cells. How these aberrant PV-positive neurons influence the differentiation of type-2 cells through GABA signaling warrants further investigation.

## 5. Conclusions

In this study, we used the *Slc6a1^S295L/S295L^* mouse model to investigate the effects of the GAT1 (S295L) mutation on the neurogenesis within the DG of the hippocampus. The quantities of type-2b intermediate progenitor cells, neuroblasts, and immature neurons were decreased in the dentate gyrus of the *Slc6a1^S295L/S295L^* mice at 4 weeks of age, indicating that GAT1 (S295L) led to impaired neurogenesis in the dentate gyrus. These abnormalities were exacerbated in adulthood, with DCX^+^ immature neurons exhibiting severe morphological impairments. The GAT1 mutation not only disrupted GABA homeostasis but also compromised the neurotrophic support essential for the proper development of the hippocampus, which could be a factor leading to neurogenesis impairment.

## Data Availability

The original contributions presented in this study are included in the article/Supplementary Materials. Further inquiries can be directed to the corresponding authors.

## Supplementary Materials

The following supporting information can be downloaded at: https://www.mdpi.com/article/10.3390/brainsci15040393/s1, Figure S1. (A–C) Representative hippocampal sections of adult WT (A), *Slc6a1^S295L/S295L^* mice (B), and *Slc6a1*^−/−^ mice (C), co-labeled with GAT1 (green) and DAPI (blue). 20×, scale bar, 100 μm. (D) DCX-positive cells in the sum of the DG compared to the male WT group (100% grey bars). (*n* ≥ 3). (E) NeuN-positive cells in the sum of the DG in comparison to the male WT group (100% grey bars). (*n* ≥ 3). Data represent mean ± S.E.M. Statistical analysis was performed using Student’s *t*-test. Figure S2. (A) Confocal images showing labeling for DCX (green) and DAPI (blue) in the DG of adult WT, *Slc6a1^S295L/S295L^* mice, and *Slc6a1*^−/−^ mice at 6 weeks (Figure above). 20×, scale bar, 100 μm. Representative DG sections of three genotypes labeled with DCX (Figure below). 100× oil, scale bar, 20 μm. (B) DCX positive cells in the DG of *Slc6a1^S295L/S295L^* mice and *Slc6a1*^−/−^ mice compared to the WT group (100% grey bars). (*n* ≥ 3) (C,D) DCX^+^ immature neurons exhibited significantly lower total dendritic length and height in *Slc6a1^S295L/S295L^* mice and *Slc6a1^−/−^* mice than in WT. (*n* ≥ 3) (E) Sholl analysis of DCX^+^ immature neurons among WT, *Slc6a1^S295L/S295L^* mice and *Slc6a1*^−/−^ mice. (*n* ≥ 3) * *p* < 0.05, ** *p* < 0.01, *** *p* < 0.001 vs. controls. Data represent mean ± S.E.M. Statistical analysis was performed using one-way ANOVA followed by Tukey’s post hoc test. Figure S3. (A) Relative expression of Bdnf in the DG of three genotypes at the age of 2 weeks, 3 weeks, 4 weeks, and 8 weeks. (*n* ≥ 4). (B) The concentration of BDNF in the hippocampus of three genotypes at the age of 4 weeks was examined by ELISA. (*n* ≥ 4). (C) The concentration of proBDNF in the hippocampus of three genotypes at the age of 4 weeks was examined by ELISA. (*n* ≥ 4). (D–F) The expression of *Gabra1*, *Gabra2*, and *Gabrg2* mRNA in the DG of WT, *Slc6a1^+/S295L^*, and *Slc6a1^S295L/S295L^* mice at 4 weeks. (*n* ≥ 3). (G–I) WB band (Top) and quantitative analysis results (bottom) of GABA_A_ receptor subunit (GABRA2, GABRA5, GABRG2) expression in the hippocampus of 4-week-old WT, *Slc6a1^+/S295L^* mice and *Slc6a1^S295L/S295L^* mice. (*n* ≥ 3). * *p* < 0.05, ** *p* < 0.01, *** *p* < 0.001 vs. controls. Data represent mean ± S.E.M. Statistical analysis was performed using one-way ANOVA followed by Tukey’s post hoc test. Figure S4. Co-localization of GAT1 (green) with parvalbumin (red) in the DG of wild type (A), *Slc6a1^+/S295L^* mice (B), and *Slc6a1^S295L/S295L^* mice (C) at 4 weeks detected by Immunohistochemistry. 100× oil, scale bar, 20 μm. The circle indicated cells that exhibited co-localization. Table S1. GO (HO vs WT); GO (HE vs HO); GO (HE vs WT).

## Abbreviations

The following abbreviations are used in this manuscript:

GABA	γ-Aminobutyric acid
GAT1	GABA transporter 1
DG	Dentate gyrus
NSCs	Neural stem cells
Sox2	SRY-box transcription factor 2
Tbr2	T-box brain protein 2
NeuroD1	Neurogenic differentiation 1
DCX	Doublecortin
NeuN	Neuronal nuclear protein
WT	Wild type
BDNF	Brain-derived neurotrophic factor
