# Supplementary material for: γ-Aminobutyric Acid Transporter Mutation GAT1 (S295L) Substantially Impairs Neurogenesis in Dentate Gyrus
Source: Brain Sci. 2025 Apr 13;15(4):393. doi: 10.3390/brainsci15040393 (PMC12025653; doi:10.3390/brainsci15040393)
Supplement: Supplementary file 1 [file brainsci-15-00393-s001.zip › brainsci-3555344-supplementary.pdf]

Supplement material

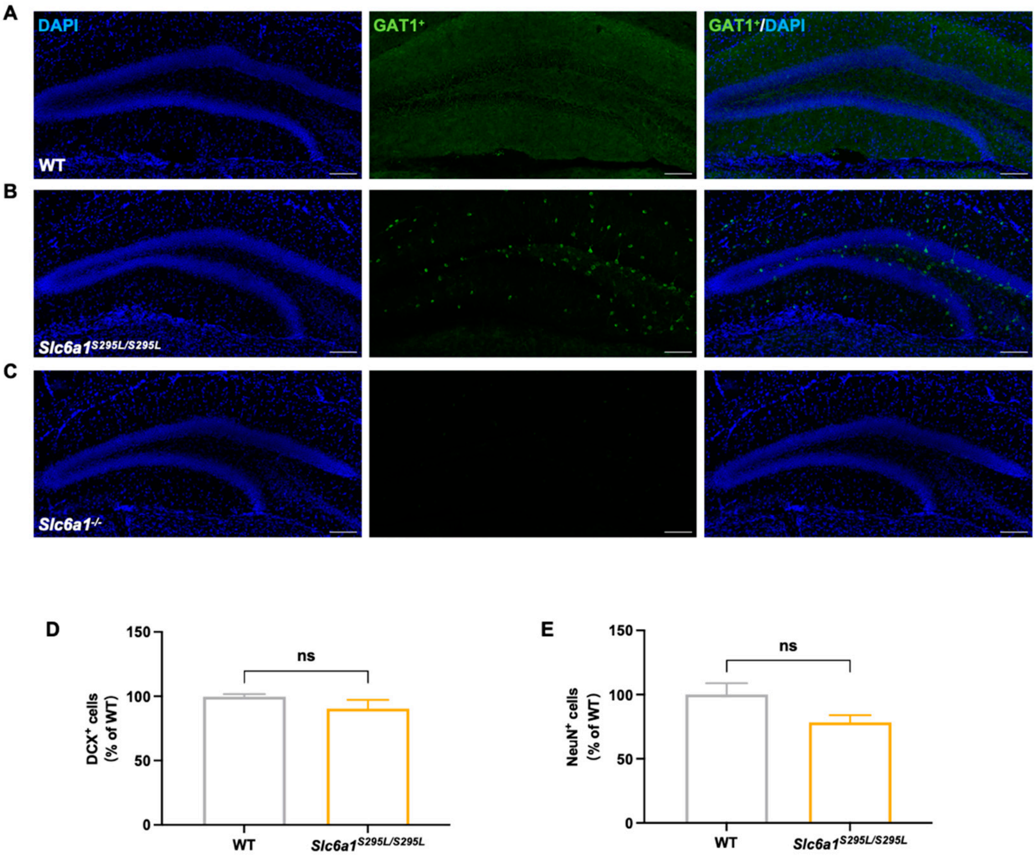

Supplementary Figure 1. (A, B, C) Representative hippocampal sections of adult WT (A), *Slc6a1*$^{S295L/S295L}$ mice (B), and *Slc6a1*$^{-/-}$ mice (C), co-labeled with GAT1 (green) and DAPI (blue). 20x, scale bar, 100µm. (D) DCX-positive cells in the sum of the DG compared to the male WT group (100% grey bars). (n≥3). (E) NeuN-positive cells in the sum of the DG in comparison to the male WT group (100% grey bars). (n≥3). Data represent mean ± S.E.M. Statistical analysis was performed using Student's t-test.

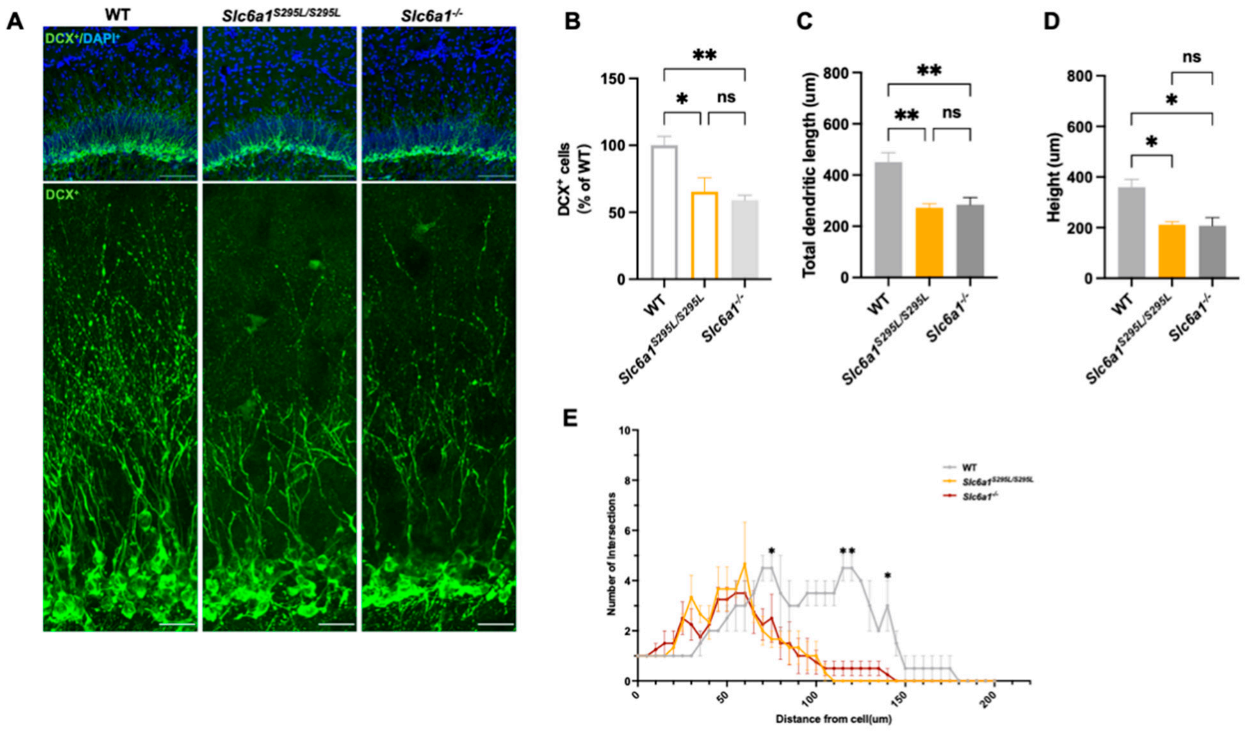

Supplementary Figure 2. (A) Confocal images showing labeling for DCX (green) and DAPI (blue) in the DG of adult WT, *Slc6a1*$^{S295L/S295L}$ mice*, and Slc6a1*$^{-/-}$ *mice at 6 weeks* (Figure above). 20x, scale bar, 100µm. Representative DG sections of three genotypes labeled with DCX (Figure below). 100x oil, scale bar, 20µm. (B) DCX positive cells in the DG of *Slc6a1*$^{S295L/S295L}$ mice and *Slc6a1*$^{-/-}$ *mice* compared to the WT group (100% grey bars). (n≥3) (C, D) DCX^{+} immature neurons exhibited significantly lower total dendritic length and height in *Slc6a1*$^{S295L/S295L}$ mice and *Slc6a1*$^{-/-}$ *mice* than in WT. (n≥3) (E) Sholl analysis of DCX^{+} immature neurons among WT, *Slc6a1*$^{S295L/S295L}$ mice and *Slc6a1*$^{-/-}$ *mice*. (n≥3) $*p < 0.05$, $**p < 0.01$, $***p < 0.001$ vs. controls. Data represent mean ± S.E.M. Statistical analysis was performed using one-way ANOVA followed by Tukey's post hoc test.

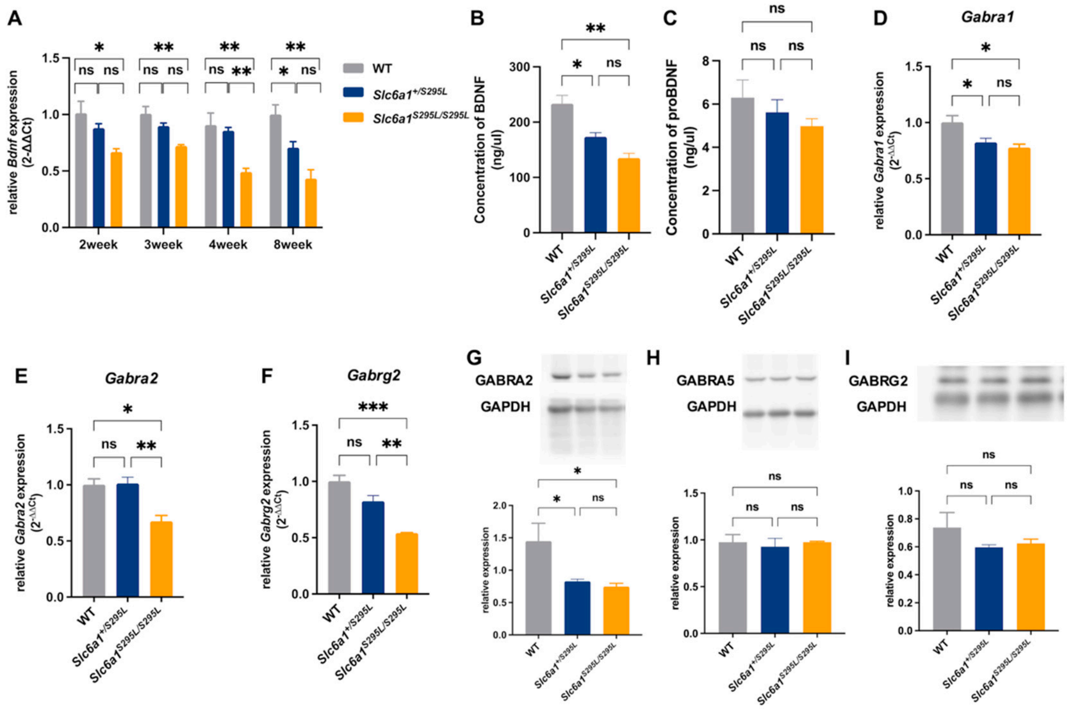

Supplementary Figure 3. (A) Relative expression of *Bdnf* in the DG of three genotypes at the age of 2 weeks, 3 weeks, 4 weeks, and 8 weeks. (n≥4). (B) The concentration of BDNF in the hippocampus of three genotypes at the age of 4 weeks was examined by ELISA. (n≥4). (C) The concentration of proBDNF in the hippocampus of three genotypes at the age of 4 weeks was examined by ELISA. (n≥4). (D-F) The expression of *Gabra1*, *Gabra2*, and *Gabrg2* mRNA in the DG of WT, *Slc6a1*$^{+/S295L}$*, and* *Slc6a1*$^{S295L/S295L}$ mice at 4 weeks. (n≥3). (G-I) WB band (Top) and quantitative analysis results (bottom) of $GABA_A$ receptor subunit (GABRA2, GABRA5, GABRG2) expression in the hippocampus of 4-week-old WT, *Slc6a1*$^{+/S295L}$ mice and *Slc6a1*$^{S295L/S295L}$ mice. (n≥3). $^{*}p < 0.05$, $^{**}p < 0.01$, $^{***}p < 0.001$ vs. controls. Data represent mean ± S.E.M. Statistical analysis was performed using one-way ANOVA followed by Tukey's post hoc test.

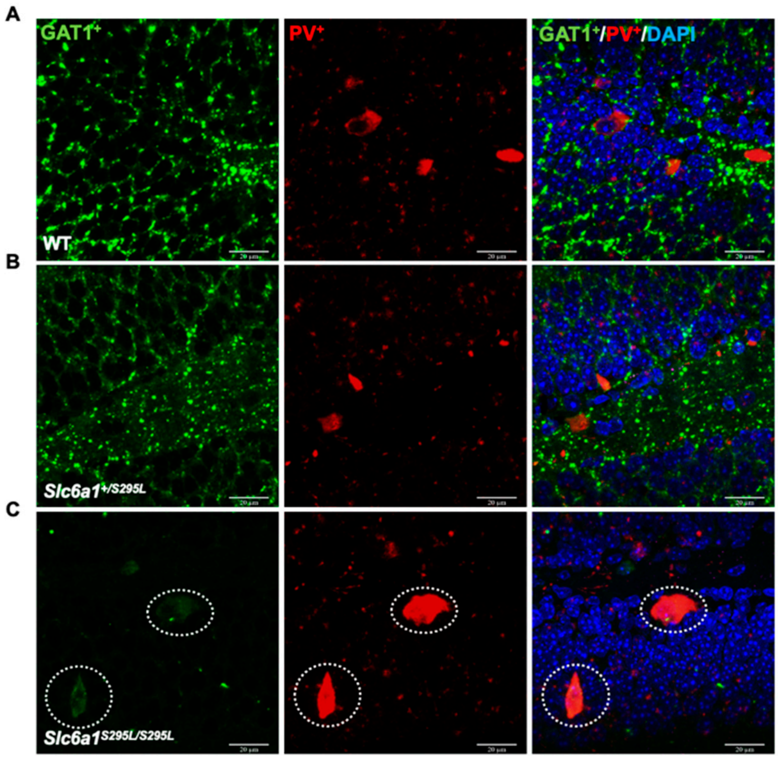

Supplementary Figure 4. Co-localization of GAT1 (green) with parvalbumin (red) in the DG of wild type (A), *Slc6a1*$^{+/S295L}$ mice (B), and *Slc6a1*$^{S295L/S295L}$ mice (C) at 4 weeks detected by **Immunohistochemistry**. 100x oil, scale bar, 20μm. The circle indicated cells that exhibited co-localization.
